# Supplementary material for: Neuropsychological Stimulation Program for Children from Low Socioeconomic Backgrounds: Study Protocol for a Randomized Controlled Trial
Source: Healthcare (Basel). 2024 Mar 6;12(5):596. doi: 10.3390/healthcare12050596 (PMC10931306; doi:10.3390/healthcare12050596)
Supplement: Supplementary file 1 [file healthcare-12-00596-s001.zip › healthcare-2880297-supplementary.pdf]

SUPPLEMENTAL MATERIALS

Comprehensive Assessment Battery

Neuropsychological assessment tests

- TOKEN Test, reduced version [36]. It measures the capacity to understand language in an order without redundancy. In this task, a series of shapes (big and small circles and squares of various colours) are laid out on the table in front of the participant, and then they will be told to touch or move them providing increasingly complicated instructions. The reduced version of the test comprises 36 items and takes approximately 10 minutes to complete. The maximum score attainable is 36, while the minimum is 0. The test-retest reliability ranges from 0.96 to 0.99.
- Verbal fluency test [37]. It measures phonological (words starting with "M") and semantic (words included in the category "animals") verbal fluency through the number of words produced by the person in each time (1 minute per condition). It takes about 4 minutes to complete. These tasks have a minimum score of 0 but don't have a stablished maximum score because it depends on how many words can a person produce in 1 minute. The ENFEN manual does not provide data on the reliability and validity of the scales that make up the battery. The manual however states that it has a Cronbach's alfa coefficient of 0.76 [Confidence interval (95%): 0.73-0.78].
- Inter-American Reading Series (L-3-DEs) [38]. This is an assessment task that can be administered in groups and measures reading proficiency of children, having subscales for vocabulary, comprehension speed and comprehension level. The version used in this study is a commercial version culturally and linguistically adapted for the Guatemalan population. It has an average duration of 60 minutes and has a maximum score of 125 and a minimum of 0. The scores of this task will be used as a screening for inclusion criteria. The participants will need to have at least a very basic ability to read and understand instructions for the program to influence them. The Inter-American Reading Series manual does not provide data on the reliability and validity, but it is a commonly used tool in the field for Latin American populations.
- Nesplora Aula School [39]. A virtual reality test for the evaluation of several abilities related to attention and self-regulation in children with an ecological

approach. In this task the participants will be sitting in a classroom, and they will have to pay attention to the blackboard and the teacher. The task will present various stimuli and tell them to which ones they must react and which ones they must ignore. The set of instructions will change throughout the task. In this project we used these main variables: 1) Attention (total omission mistakes), 2) Inhibitory Control (total commission mistakes), 3) Impulsivity (mean reaction time while committing mistakes), and 4) Reaction speed (mean reaction time when emitting correct responses). It has an average duration of 15 minutes, and the results are given directly as percentiles scores depending on the participant's age. The test-retest reliability ranges from 0.96 to 0.98.

- Nesplora Ice Cream [40]. A virtual reality test for the evaluation of Executive Functions. In this task, people will have to attend customers in an ice cream shop, giving priority to the orders depending on how the customer is dressed. Customers will order different flavors and it is up to the participant to give them the correct one from a recipe book that changes mid-task. We used the main variables: 1) Working Memory (mean between total amount of correct responses and total processing speed), 2) Planification (mean between number of turns assigned and the average round duration), 3) Cognitive Flexibility (mean between the task interference -difference between correct responses in the two halves of the task-, perseverative behaviour -number of incorrect responses during the second half of the task, which were correct in the first half-, and switching -time increment between turns-), and 4) Learning interference (increment of recipe book checking in the second part of the task). It has an average duration of 30 minutes, and the results are given directly as percentiles scores depending on the participant's age. The test-retest reliability ranges from 0.85 to 0.97.
- Non-verbal Intelligence Test (TONI-2) [41]. It was developed as an intelligence measurement free of language influence, motor skills or reading capabilities, looking only for reasoning skills to solve abstract puzzles. It consists of 55 items of increasing difficulty, lasting approximately between 15-20 minutes. It has a maximum score of 55 and a minimum of 0. The scores of this task will be also used as a screening for inclusion criteria. The test-retest reliability of 0.89 (Form A) and 0.83 (Form B).

- The Probabilistic Decision-Making Task [42] measures the risk aversion of children utilizing a Multiple Prices List [43]. To facilitate its comprehension by children, the number of decisions has been changed from 10 to 6 and the lotteries are represented as classic gumball machines. The risk aversion score is the number of safer options children make (between 0 to 6). Risk aversion has a correlation with human capital, entrepreneurship, and other socioeconomic variables. It has a duration of approximately 3 minutes. The authors do not report a reliability measure.
- The Temporal Discount Task [42]. It measures the ability of children to discount a gain or a prize that isn't immediate (delayed in time). This task is an adaptation from the original [44] that includes pictures and reduces the number of items from 11 to 6 to facilitate its comprehension by children. The number of times that children choose the delayed option (from 0 to 6) is the measurement of Patience (ability to delay gratification), which correlates with education, income, savings, and health. It has a duration of approximately 3 minutes. The authors do not report a reliability measure.
- NESPY-II Assessment Battery [45]. This exhaustive neuropsychological battery is designed for the cognitive assessment of children and adolescents. In this project we will use two subscales directly related to Social Cognition skills: 1) Emotion Recognition, and 2) Theory of Mind. The task Emotion Recognition measures the ability of children to correctly identify emotions in pictures of other children. It has 35 items and has a duration of about 5 minutes, with a maximum score of 35 and a minimum of 0. The Theory of mind task measures the ability of children to interpret and understand beliefs, intentions, faking and lies, emotions, and imagination in other people. It also measures if kids can understand social context and its relationship with emotions. It consists of 21 items and has a duration of about 5 minutes, with a maximum score of 28 and a minimum of 0. The test-retest reliability ranges from 0.66 to 0.84.

Emotional management, Mental Health, and Quality of Life questionnaires

- The Childhood Depression Inventory, CDI [46]. In this study we will use the reduced 10 items version adapted to the Spanish language. It measures depressive symptoms in children and adolescents with 10 items divided in five subscales: negative mood, inefficacy, low self-esteem, social withdrawal, and

pessimism. Each item has a score from 0 to 2 depending on the severity. It has a duration of approximately 5 minutes with a maximum score of 20 and a minimum of 0. The test-retest reliability ranges from 0.77 to 0.89.

- The Spence Children Anxiety Scale, SCAS [47], assess frequent anxiety symptoms in children divided into 6 subscales: Panic attacks/agoraphobia, separation anxiety, social phobia, general fears, generalised anxiety, and obsessions/compulsions. In this study we will use a condensed 12 items version adapted to the Spanish language focused only in two subscales: generalised anxiety and separation anxiety. Each item has a score from 0 to 3 depending on its frequency. It has an approximate duration of 5 minutes with a maximum score of 36 and a minimum of 0. The test-retest reliability ranges from 0.48 to 0.81.
- The Multidimensional School Anger Inventory, MSAI [48]. In this study we will use a reduced 12 items version adapted to the Spanish language. It has three different subscales that measure anger experience, hostility, and destructive expression /anger management, in children and adolescents. Each item has a score from 1 to 4 depending on the level of emotional response of the child to the situation. It has an approximate duration of 5 minutes with a maximum score of 12 and a minimum of 4 for each of the 3 subscales. The test-retest reliability ranges from 0.56 to 0.62.
- The Health-related quality of life scale, KIDSCREEN-10 [49]. In this study we will use the 10 items version of this scale adapted to the Spanish language. It asks the children various questions related to the subjective perception of their own physical, mental, and social health, each item has a score between 1 and 5. It has an approximate duration of 5 minutes with a maximum score of 50 and a minimum of 10. The test-retest reliability of 0.73.

Socio-economic, clinical, and academic assessment

- A sociodemographic questionnaire will be handed to families to give out information such as: names, age, occupation, and mean educational level of legal guardians (MLPE), number of people that live in the family household, or monthly income of the family household in Quetzals (Q).
- A clinical questionnaire will be administered asking if there were any health problems for the mother or child during pregnancy or childbirth, if the child

needed to be in an incubator, if the child had any clinical diagnostic, and if it was or is currently under any pharmacological treatment.

- A language experience questionnaire that will assess the languages that both the legal guardians and the children can understand and speak, including their degree of proficiency.
- The previous school year marks of children will be recorded to stablish a base line for academic performance. To include all knowledge disciplines, an average score of all subjects will be used as the “average marks” variable. An average of all subjects at the end of this academic year will be calculated as the post-intervention variable. The subjects imparted at Fe y Alegría Guatemala schools are communication and language, mathematics, natural sciences, social sciences, artistic expression, physical education, civic education, productivity and development, and musical education.
- The Latin-American and Caribbean Latin-American Food Security Scale (ELCSA) [50], validated by the Food and Agriculture Organization of the United Nations. This scale is composed of two different parts answered by the caregivers. First, caregivers are asked how safe they feel in relation to the food they can provide to the family. The assessment of this part offered a score between 1 to 15 in which higher scores reflect higher food insecurity. The second section asks how many days per week the family consumes a certain type of food like vegetables, meat and fish, sugary foods, etc. The test-retest reliability ranges from 0.92 to 0.95.
- The VEX-R PLUS Self-Reported Violence Assessment Questionnaire [51]. Created to measure the exposure of children to violence, it has a version for children and parents. In this study we will only use the children version that has a total of 30 items (4 neutral examples and 26 violent situations) covering many types of violence plus an extra 4 covering sexual violence. Each item has a score between 0 and 4 depending on the level of exposure, having a maximum score of 104 and a minimum of 0. The test-retest reliability ranges from 0.72 to 0.86.
